# Plant cell wall dynamics and wall-related susceptibility in plant–pathogen interactions

**DOI:** 10.3389/fpls.2014.00228

**Published:** 2014-05-28

**Authors:** Daniela Bellincampi, Felice Cervone, Vincenzo Lionetti

**Affiliations:** Dipartimento di Biologia e Biotecnologie “Charles Darwin”, Sapienza Università di RomaRome, Italy

**Keywords:** cell wall, cell wall integrity, host cell wall metabolism reprogramming, plant defense, susceptibility factors

## Abstract

The cell wall is a dynamic structure that often determines the outcome of the interactions between plants and pathogens. It is a barrier that pathogens need to breach to colonize the plant tissue. While fungal necrotrophs extensively destroy the integrity of the cell wall through the combined action of degrading enzymes, biotrophic fungi require a more localized and controlled degradation of the cell wall in order to keep the host cells alive and utilize their feeding structures. Also bacteria and nematodes need to degrade the plant cell wall at a certain stage of their infection process, to obtain nutrients for their growth. Plants have developed a system for sensing pathogens and monitoring the cell wall integrity, upon which they activate defense responses that lead to a dynamic cell wall remodeling required to prevent the disease. Pathogens, on the other hand, may exploit the host cell wall metabolism to support the infection. We review here the strategies utilized by both plants and pathogens to prevail in the cell wall battleground.

## INTRODUCTION

Phytopathogenic fungi, bacteria, and nematodes infect, grow and reproduce themselves on the plant tissues and, at least at the early stages of infection, require breaking the integrity of the host cell wall. Beyond the cuticle layer, the interaction with the plant cell wall and the extent of the wall degradation are determined by the lifestyle of the pathogen. Plants perceive a diverse set of microbial molecules referred to as microbial/pathogen associated molecular patterns (MAMPs/PAMPs; Boller and He, 2009) through high-affinity cell surface pattern recognition receptors (PRRs) leading to intracellular signaling, transcriptional reprogramming, and biosynthesis of defense metabolites that limit the microbial infection (Dangl et al., 2013). Emerging evidences indicate that plant cells also exploit sophisticated mechanisms of sensing the alteration of cell wall integrity (CWI) during biotic stress (Hamann, 2012; Pogorelko et al., 2013a). For instance, they perceive endogenous molecules produced in damaged tissues (the so-called damage-associated molecular patterns, or DAMPs) through membrane receptors (Ferrari et al., 2013). The loss of CWI induced by pathogens activates a variety of defense responses including a cell wall remodeling required to prevent the disease. To escape recognition, pathogens produce effector proteins that counteract the plant defenses (Giraldo and Valent, 2013) and, sometimes, exploit the host cell wall metabolism to favor the infection process (Cantu et al., 2008b).

## CELL WALL DYNAMICS DURING INFECTION BY MICROBIAL PATHOGENS

**FIGURE 1:**
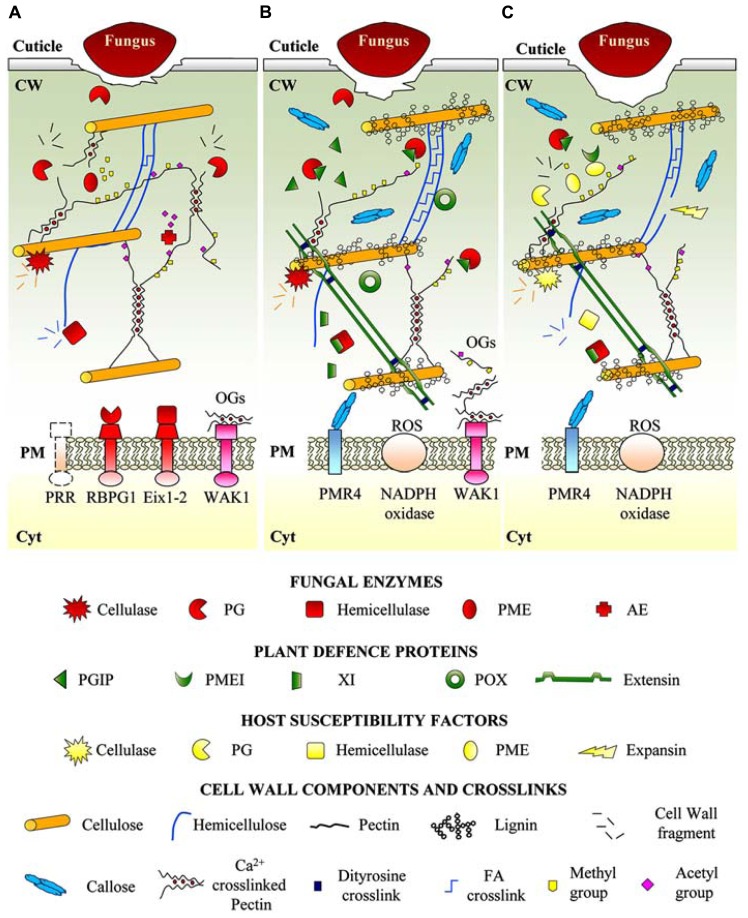
**Cell wall dynamics during necrotrophs invasion. (A)** Necrotrophic fungi secrete a large arsenal of cell wall degrading enzymes (CWDEs) like PGs, hemicellulases and cellulases, assisted by PMEs and AEs in the apoplastic space to degrade cell wall polymers and facilitate the availability of nutrients. PGs and EIXs have been proposed to function as PAMPs recognized by the membrane receptors RBPG1 and Eix1 or 2, respectively. **(B)** As first line of defense, plants produce a variety of CWDE inhibitors to hinder degradation by microbial CWDEs. For instance, the inhibition of PG degrading activity by PGIPs induces the accumulation of elicitor-active pectin fragments (OGs) perceived by WAK1 receptors. It cannot be excluded the presence of other not yet identified receptors sensing damage of other cell wall components. The perception of cell wall damage triggers specific signaling pathways activating defense responces aimed to reinforce cell wall structure. The more evident defense strategies are callose and lignin deposition, induction of peroxidases/ROS-mediated crosslinks between cell wall structural proteins and polysaccharides. **(C)** Necrotrophs force plants to cooperate in disease exploiting plant cellulases, expansins, PGs and PMEs as susceptibility factors. PM, plasma membrane; CW, cell wall; Cyt, cytoplasm; OGs, oligogalacturonides; WAK1, wall associated kinase 1; AEs, acetyl esterases; PGs, polygalacturonases; EIXs, ethylen induced xylanases; PME, pectin methylesterases; PMEI, pectin methylesterase inhibitor; FA, ferulic acid; Eix1-2, receptors of ethylene induced xylanases; RBPG1, Responsiveness to Botrytis PolyGalacturonase 1; Ca^2+^, calcium ions; XI, xylanase inhibitor; PRR, pattern recognition receptor; POX, peroxidase; ROS, reactive oxygen species.

Infection by fungal necrotrophs is a complex process that includes conidial attachment, germination, host penetration, lesion formation and expansion, and tissue maceration followed by sporulation (Prins et al., 2000). Penetration may be achieved by degrading the external cuticle through the action of cutinases and lipases (Laluk and Mengiste, 2010). The role of the cuticle in plant defense is discussed elsewhere (Chassot and Metraux, 2005; Chassot et al., 2007). Once penetrated the cuticle, necrotrophs have a spatial and temporal strategy of attacking the plant cell wall by producing several cell wall degrading enzymes (CWDEs) belonging to multiple families (**Figure 1A**). The diversity of these enzymes mirrors the structural complexity and the dynamics of the cell wall as well as the lifestyle and host adaptation of the pathogen (King et al., 2011). The extensive degradation of cell wall polysaccharides by necrotrophs is sensed by plants. The leucine-rich repeat receptor-like kinase (LRR-RLK) ERECTA (ER) and the heterotrimeric G-protein are involved in cell wall remodeling during *Arabidopsis* defense response against *Plectosphaerella cucumerina* and probably control CWI (Llorente et al., 2005; Sanchez-Rodriguez et al., 2009). The impairment of cellulose synthases involved in secondary cell wall deposition is also a mechanism of sensing CWI and enhances disease resistance of *Arabidopsis* to *P. cucumerina* and *Ralstonia solanacearum* (Hernandez-Blanco et al., 2007).

One of the strategies used by plants to limit the degradation of the cell wall polysaccharides by microbial CWDEs is the production of proteinaceous inhibitors (**Figures 1A**, B). Polygalacturonases (PGs) are pathogenicity factors produced at the earlier stages of a microbial infection that depolymerize the homogalacturonan (HG), i.e., the main component of pectin in dicots but also present in monocots (Caprari et al., 1993; D’Ovidio et al., 2004). Against microbial and insect PGs, plants produce cell wall-associated polygalacturonase-inhibiting proteins (PGIPs; Spadoni et al., 2006). The over expression of PGIPs improves the resistance to fungal and bacterial necrotrophs in different plants (Aguero et al., 2005; Ferrari et al., 2012). The PG-PGIP interaction results in the accumulation of elicitor-active oligogalacturonides (OGs) that are perceived in *Arabidopsis* by the receptor Wall Associated Kinase 1 (WAK1; Brutus et al., 2010) to activate the plant immune responses (Ferrari et al., 2013). Accumulation and sensing of OGs in response to pathogens is critical for monitoring the pectin integrity and, in general, a tissue injury (De Lorenzo et al., 2011). Alteration of pectin integrity caused by the expression of PGII from *Aspergillus niger* in tobacco and *Arabidopsis* causes a constitutive activation of defense genes and resistance against *Botrytis cinerea* (Ferrari et al., 2008). Recently, *B. cinerea* and *A. niger* PGs have been proposed to function themselves as PAMPs recognized by the *Arabidopsis* Receptor-Like Responsiveness to Botrytis PolyGalacturonase 1 (RBPG1) belonging to a super clade of LRR receptor-like proteins (RLPs; Zhang et al., 2014).

Xylan is the major hemicellulose polymer in cereals. To counteract xylan degradation by microbial endoxylanases, graminaceous monocots produce the *Triticum aestivum* xylanase inhibitor (TAXI), the xylanase inhibitor protein (XIP) and the thaumatin-like xylanase inhibitor (TL-XI; Bellincampi et al., 2004; Juge, 2006). The constitutive expression of TAXI-III in wheat reduces susceptibility to *Fusarium graminearum* (Moscetti et al., 2013). On the other hand, fungal xylanases function as PAMPs by eliciting defense responses and promoting necrosis (Noda et al., 2010; Sella et al., 2013). Ethylene inducing xylanases (EIXs) produced by *Trichoderma* species are perceived in tomato, by two specific LRR-RLPs receptors, LeEix1 and LeEix2 (Ron and Avni, 2004). Both receptors bind Eixs, while only LeEix2 mediates defense responses. LeEix1 heterodimerizes with LeEix2 upon application of the Eixs and attenuates Eix-induced internalization and signaling of the LeEix2 receptor (Bar et al., 2010). Xyloglucan, i.e., the main hemicellulosic polysaccharide in the primary walls of dicots and non-graminaceous monocots, is degraded by microbial xyloglucan-specific endoglucanases (XEGs). Fungal XEGs are inhibited by xyloglucan endoglucanase inhibiting proteins (XEGIPs), which so far have been characterized in tomato, carrot and tobacco (Juge, 2006).

Reinforcement of the cell wall is initiated at the pathogen penetration sites in response to cell wall damage (**Figure 1B**). Deposition of callose by the callose synthase PMR4 occurs upon infection of *Arabidopsis* with *P. cucumerina* and *Alternaria brassicicola* (Ton and Mauch-Mani, 2004; Flors et al., 2008). Callose deposition is triggered by PAMPs and DAMPs, is affected by environmental conditions and requires the apoplastic accumulation of the hydrolysis products of glucosinolates or benzoxazinoid metabolites (Galletti et al., 2008; Ahmad et al., 2011; Luna et al., 2011).

Deposition of lignin has been associated to resistance of cotton to *Verticillium dahliae* and of *Camelina sativa* to *Sclerotinia sclerotiorum* (Xu et al., 2011; Eynck et al., 2012). Lignin makes the cell wall more resistant to CWDEs and also prevents the diffusion of pathogen-produced toxins (Sattler and Funnell-Harris, 2013). The cell wall may also be reinforced by cross-links and insolubilization of structural proteins like the hydroxyproline-rich glycoproteins (HRGPs) by peroxidase-mediated isodityrosine linkages formed in response to pathogen attach (Deepak et al., 2010). Plant peroxidases catalyze cross-links between phenolic compounds in the secondary walls and between polysaccharides and ferulic acid (FA) upon attack by necrotrophs (Passardi et al., 2004). Crosslinks between FA and polysaccharides enhance the recalcitrance of the cell wall to digestion by microbial CWDEs and the overall resistance to fungi (Bily et al., 2003). On the other hand, fungal FA esterases may shear FA from the cell wall polysaccharides (Udatha et al., 2012).

The activities of pectin methyl esterases (PMEs) from both plants and pathogens and the degree and pattern of pectin methyl esterification are critical for the outcome of plant–pathogen infections (Lionetti et al., 2012). The cell walls containing highly methylesterified pectin are somewhat protected against the action of microbial PGs and pectate lyases (PLs; Arancibia and Motsenbocker, 2006). PMEs, which remove methyl esters from pectin, are controlled by PME inhibitor proteins (PMEIs) either during growth and development (Raiola et al., 2004; Rocchi et al., 2011; Reca et al., 2012) and during plant–pathogen interactions (Lionetti et al., 2012). The biochemical and structural bases of the enzyme/inhibitor interaction have been elucidated (Mattei et al., 2002; Di Matteo et al., 2005). *Arabidopsis* over expressing PMEIs have a lower level of PME activity, a higher degree of pectin esterification and a concomitant reduced susceptibility to *B. cinerea* and *Pectobacterium carotovorum* (Lionetti et al., 2007; Raiola et al., 2011). The ectopic expression in wheat of a PME inhibitor from kiwi reduces the susceptibility to *F. graminearum* and *Bipolaris sorokiniana* (Volpi et al., 2011). The transcription factor MYB46 which affect the secondary cell wall biosynthesis (Zhong et al., 2007), regulates the expression of genes encoding several cell wall proteins including PMEI and mediates disease susceptibility of *Arabidopsis* to *B. cinerea* (Ramirez et al., 2011). Recently, jasmonic acid has been proposed to modulate the degree of methylesterification in potato to protect pectin degradation by PLs produced by *Dickeya dadantii* (Taurino et al., 2014). Acetylation of the cell wall polysaccharides is also a determinant of plant–pathogen interaction. An *Arabidopsis* mutant with reduced acetylation displays increased tolerance to *B. cinerea* (Manabe et al., 2011). *Arabidopsis* and *Brachypodium distachyon* plants expressing xylan or pectin acetylesterases from *A. nidulans* activate specific defense responses and are more resistant to *B. cinerea* and *B. sorokiniana* (Pogorelko et al., 2013b).

**FIGURE 2:**
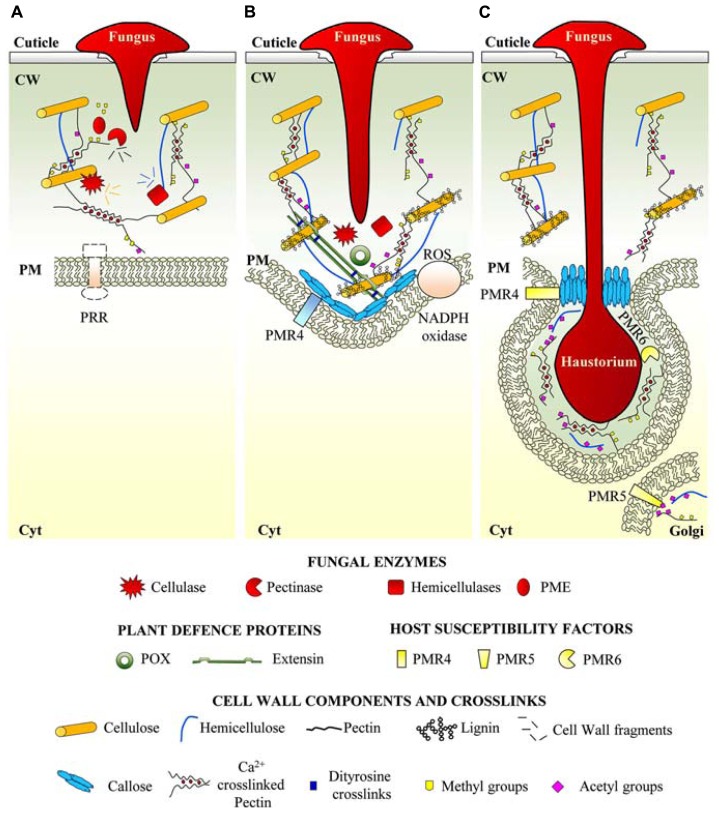
**Cell wall dynamics in plant-biotrophic fungi interaction. (A)** Biotrophic fungi use appressorial mechanical pressure and secrete cell wall degrading enzymes to penetrate plant cell wall. **(B)** Plants perceive fungal biotrophs penetration with not yet identified receptors and respond with “papillae” apposition between cell wall and plasma membrane. Papillae, in addition to new cell wall material are also sites of accumulation of ROS possibly involved in cell wall reinforcement. **(C)** At a later stage of infection, fungus forms the haustorium feeding organ invaginated into the host membranes and plant cell wall. Biotrophs locally affect cell wall metabolism by induction of susceptibility factors (callose synthase PMR4, *O*-acetyltransferase PMR5 and pectate lyase PMR6) to modify the extrahaustorial matrix to improve the accessibility of nutrients or to ensure the mechanical stability of the haustorium. PM, plasma membrane; CW, cell wall; Cyt, cytoplasm; PG, polygalacturonase; PME, pectin methylesterase; PRR, pattern recognition receptor; POX, peroxidase; ROS, reactive oxygen species.

Biotrophic and hemi-biotrophic fungi acquire nutrients from the host cells without causing their death. They often attack the plant surface and penetrate the external barriers by developing appressoria and exploiting the mechanical pressure (Wilson and Talbot, 2009). In order to breach the host cuticle they also secrete oxidases, esterases, cutinases, and lipases (Feng et al., 2011). Small amounts of CWDEs associated with local softening and loosening of plant cell walls are produced by biotrophic microorganisms (Zhao et al., 2013) (**Figure 2A**). Plants contrast invasion of biotrophs by the apposition of “papillae,” cell wall thickening early produced at the site of pathogen penetration (**Figure 2B**). Papillae contain callose, as most abundant constituent, cellulose, hemicelluloses, pectins, lignin, and structural proteins such as arabinogalactan proteins and HRGPs (Aist, 1976; Celio et al., 2004; Voigt, 2014). Transgenic *Arabidopsis* plants overexpressing the callose synthase PMR4 show an early and elevated deposition of callose at the sites of penetration which prevents the haustoria formation and further penetration by *Golovinomyces cichoracearum* and *Blumeria graminis* (Ellinger et al., 2013). Papillae are also the sites where antimicrobial peptides, toxic secondary metabolites and reactive oxygen species (ROS) accumulate and contribute to plant resistance (Bednarek et al., 2009; Daudi et al., 2012). Lignification and cross-links of proteins in the papillar cell wall may entrap the penetration peg of biotrophic fungi and render the cell wall more resistant to the mechanical pressure exerted by fungal appressoria (Bechinger et al., 1999; O’Brien et al., 2012). Lignin downregulation may also activate defense responses and increases the resistance to the hemibiotroph *Colletotrichum trifolii* in alfalfa (*Medicago sativa* L.) (Gallego-Giraldo et al., 2011).

A reduced cellulose content by mutation of cellulose synthase *CESA3*, involved in primary cell wall formation, leads to production of lignin, and makes *Arabidopsis* more resistant to different powdery mildew pathogens (Ellis and Turner, 2001; Ellis et al., 2002; Cano-Delgado et al., 2003). RLKs belonging to the *Catharanthus roseus* (CrRLK)-like protein family are implicated in CWI mechanisms. Among these, THESEUS1 (THE1) is required for lignification in response to inhibition of cellulose biosynthesis (Hematy et al., 2007).

Alteration of pectin integrity can trigger plant immunity also against hemibiotrophs (Ferrari et al., 2008; Bethke et al., 2013). *Arabidopsis* PMEs, triggered by a Jasmonic acid dependent pathway, contribute to plant immunity against *Pseudomonas syringae*(Bethke et al., 2013).

## MICROBIAL PATHOGENS EXPLOIT THE HOST CELL WALL METABOLISM TO FACILITATE PATHOGENESIS

Necrotrophs can force plants to cooperate in disease by altering the host cell wall and favoring the cell wall accessibility to CWDEs (Hok et al., 2010; **Figure 1C**). The *Arabidopsis* AtPME3 is induced upon infection with *B. cinerea* and *P. carotovorum* and functions as susceptibility factor required for the initial colonization of the host tissue (Raiola et al., 2011). A PG (LePG) and expansin (LeExp1) cooperatively contribute to cell wall loosening during tomato ripening; their expression is induced by necrotrophic pathogens to successfully infect fruits (Cantu et al., 2009). Transgenic tomato fruits with suppressed expression of LePG and LeExp1 exhibit a reduced susceptibility to *B. cinerea* (Cantu et al., 2008a). Silencing of two putative endo β-1,4-endoglucanases, involved in the hydrolysis of cellulose or hemicellulose during ripening, cause a reduced susceptibility of tomato fruits to *B. cinerea* (Flors et al., 2007).

Biotrophic fungi, at a later stage of infection, produce a limited and localized degradation of the cell wall in the epidermal or mesophyll cells (Herbert et al., 2004). On the other hand, they form intracellular structures, like the “haustoria,” i.e., feeding organs invaginated into the host membranes to acquire nutrients. Biotrophs need to avoid the host defense responses and carefully regulate the cell-wall degradation at the border of their feeding structures to allow fungal accommodation and haustorium function (**Figure 2C**). A screening for *Arabidopsis* powdery mildew-resistant mutants allowed isolating two pectin-related genes, *PMR5* and *PMR6* which are pathogen-induced and required for susceptibility to *G. cichoracearum* and *G. orontii* (Vogel et al., 2002, 2004). *PMR5* encodes a protein with unknown function that shares sequence similarity with genes encoding polysaccharide *O*-acetyltransferase (Gille and Pauly, 2012). Therefore, acetylation may be a host susceptibility mechanism that is reprogrammed by biotrophs during infection. PMR6 encodes a putative PL that is, possibly, recruited by the fungi as a susceptible factor to reduce Ca^+^^+^-pectate domains at the level of haustoria-plasma membrane and facilitate cell wall porosity and accessibility of host nutrients (Vogel et al., 2002). Callose deposition may also work in favor of the pathogen by contributing to the stability and function of the haustoria and acting as a barrier that renders haustoria less susceptible to toxic metabolites that are produced by the host and accumulate in the site of infection (Jacobs et al., 2003). On the other hand, callose may limit the diffusion of pathogen-derived elicitors, thus reducing the activation of defense responses (Underwood, 2012).

Many bacterial pathogens utilize a type III secretion system to inject effector proteins directly into the host cytoplasm and manipulate the host cellular activities to their own advantage (Buttner and He, 2009). The effector AvrPto of *P. syringae* suppresses a set of *Arabidopsis* genes that encode cell wall-related defense proteins such as HRGPs (Hauck et al., 2003).

## CELL WALL DYNAMICS IN PLANT INTERACTIONS WITH NEMATODES AND VIRUSES

Changes in the cell wall metabolism occur during plant infection by nematodes (Barcala et al., 2010; Bohlmann and Sobczak, 2014). Like biotrophic pathogens, root-knot and cyst nematodes need to establish feeding structures inside the plant tissue to allow the uptake of nutrients (Davis et al., 2004; Williamson and Kumar, 2006). This process is assisted by the secretion of CWDEs such as pectinases and cellulases produced by the nematodes, (Vanholme et al., 2004; Davis et al., 2008) and by the local expression of host proteins like expansins and cellulases (Wieczorek et al., 2006, 2008). The sugar beet cyst nematode *Heterodera schactii* infects *Arabidopsis* roots and exploits the host-encoded AtPME3. Transgenic plants overexpressing AtPME3 exhibit an increased susceptibility to the nematode. It has been proposed that AtPME3 locally reduces the pectin esterification and improves the cell wall loosening of pre-syncytial cells during the early stages of syncytium formation (Hewezi et al., 2008).

Callose deposition at the level of plasmodesmata (PD) limits the cell-to-cell spreading of plant viruses. Due to the small diameter of the PD pore, some viruses utilize the viral movement proteins (MPs) to modify the PD size exclusion limit. Specific interactions of viral MPs with PME are often required (Chen et al., 2000). In addition to MP-PME interaction, the PME-dependent formation of methanol has also been reported to be important for viral cell-to-cell movement (Dorokhov et al., 2012; Komarova et al., 2014). The overexpression of PME inhibitor proteins in tobacco and *Arabidopsis* contrasts the cell-to-cell and systemic movement of tobamoviruses (Lionetti et al., 2013).

## CONCLUSION

The cell wall is the battleground where plants and pathogens attempt to prevail by implementing contrasting wall-reinforcing and wall-weakening strategies. When pathogens start degrading the plant cell wall components, plants are capable of perceiving the loss of wall integrity and subsequently activate the defense signaling pathways. Pathogens try to escape the plant defenses and sometimes take advantage of the host cell wall metabolism to facilitate their entry into the tissue. These dynamic processes vary according to the lifestyle of the pathogen and the type of plant pathogen interaction. While necrotrophy involves a strong and diffused molecular warfare that may provoke extended lesions of the tissue, during biotrophy the battle involves a weaker cell wall degradation mainly localized at the point of penetration and at the level of the feeding apparatus. Perception of cell wall damage as well as the pathogen- and host-induced cell wall remodeling occurs in both cases. The damage of specific cell wall polysaccharides during infection may be perceived by receptors as THE1, ER and WAK1. Plants may also rely on the recognition of CWDEs by LRR-RLPs receptors, as RBPG1 and LeEIX1-2. Cell wall fragments may be released during infection and sensed as damage signals. Analysis of cell wall mutants has shed light on the relationship between cell wall remodeling and plant response to pathogens. The expression of endogenous and microbial CWDEs and their inhibitors is also a valuable approach for studying the dynamics of the cell wall during plant–pathogen interactions as well as a strategy to improve plant protection.

## Conflict of Interest Statement

The Review Editor, Dr Ferrari, declares that, despite being affiliated to the same institution as authors Dr Daniela Bellincampi and Dr Felice Cervone, the review process was handled objectively. The authors declare that the research was conducted in the absence of any commercial or financial relationships that could be construed as a potential conflict of interest.
